# Excess multi-cause mortality linked to influenza virus infection in China, 2012–2021: a population-based study

**DOI:** 10.3389/fpubh.2024.1399672

**Published:** 2024-05-30

**Authors:** Tian-Lu Yin, Ning Chen, Jin-Yao Zhang, Shuang Yang, Wei-Min Li, Xiao-Huan Gao, Hao-Lin Shi, Hong-Pu Hu

**Affiliations:** ^1^Institute of Medical Information, Chinese Academy of Medical Sciences & Peking Union Medical College, Beijing, China; ^2^School of Public Health, Shanghai Jiaotong University School of Medicine, Shanghai, China; ^3^Tuberculosis and Thoracic Tumor Research Institute, Beijing Chest Hospital, Beijing, China; ^4^Medical College, Hebei Engineering University, Hebei, China

**Keywords:** influenza, burden of disease, China, excess mortality, negative binomial regression model, epidemic disease, prevention strategy

## Abstract

**Objectives:**

The aim of this study is to estimate the excess mortality burden of influenza virus infection in China from 2012 to 2021, with a concurrent analysis of its associated disease manifestations.

**Methods:**

Laboratory surveillance data on influenza, relevant population demographics, and mortality records, including cause of death data in China, spanning the years 2012 to 2021, were incorporated into a comprehensive analysis. A negative binomial regression model was utilized to calculate the excess mortality rate associated with influenza, taking into consideration factors such as year, subtype, and cause of death.

**Results:**

There was no evidence to indicate a correlation between malignant neoplasms and any subtype of influenza, despite the examination of the effect of influenza on the mortality burden of eight diseases. A total of 327,520 samples testing positive for influenza virus were isolated between 2012 and 2021, with a significant decrease in the positivity rate observed during the periods of 2012–2013 and 2019–2020. China experienced an average annual influenza-associated excess deaths of 201721.78 and an average annual excess mortality rate of 14.53 per 100,000 people during the research period. Among the causes of mortality that were examined, respiratory and circulatory diseases (R&C) accounted for the most significant proportion (58.50%). Fatalities attributed to respiratory and circulatory diseases exhibited discernible temporal patterns, whereas deaths attributable to other causes were dispersed over the course of the year.

**Conclusion:**

Theoretically, the contribution of these disease types to excess influenza-related fatalities can serve as a foundation for early warning and targeted influenza surveillance. Additionally, it is possible to assess the costs of prevention and control measures and the public health repercussions of epidemics with greater precision.

## 1 Introduction

Influenza, commonly known as the flu, is the foremost infectious disease subject to global surveillance (1), highlighting its detrimental effects. An estimated 290,000 to 650,000 deaths occur annually as a result of seasonal or pandemic influenza, with a particularly profound impact on older adults, according to global data (2). Accurate information regarding the burden of influenza-associated diseases is pivotal for effective immunization strategies and the allocation of public health resources. However, official statistics frequently fail to provide an accurate depiction of the true incidence and mortality rates associated with influenza due to inadequate recognition of primary influenza infections and delayed reporting of severe clinical outcomes resulting in the cause of death being classified as due to other diseases. Additionally, evaluating the impact of social determinants on disease burden during influenza outbreaks poses challenges (3). Mathematical models are frequently used on a global scale to compute influenza-associated excess mortality rates during influenza seasons. These models calculate the proportion of deaths that exceed baseline levels and serve as a metric to assess the societal and health repercussions of influenza, with a particular focus on the impact of the epidemic of infectious diseases (4).

**Figure 1:**
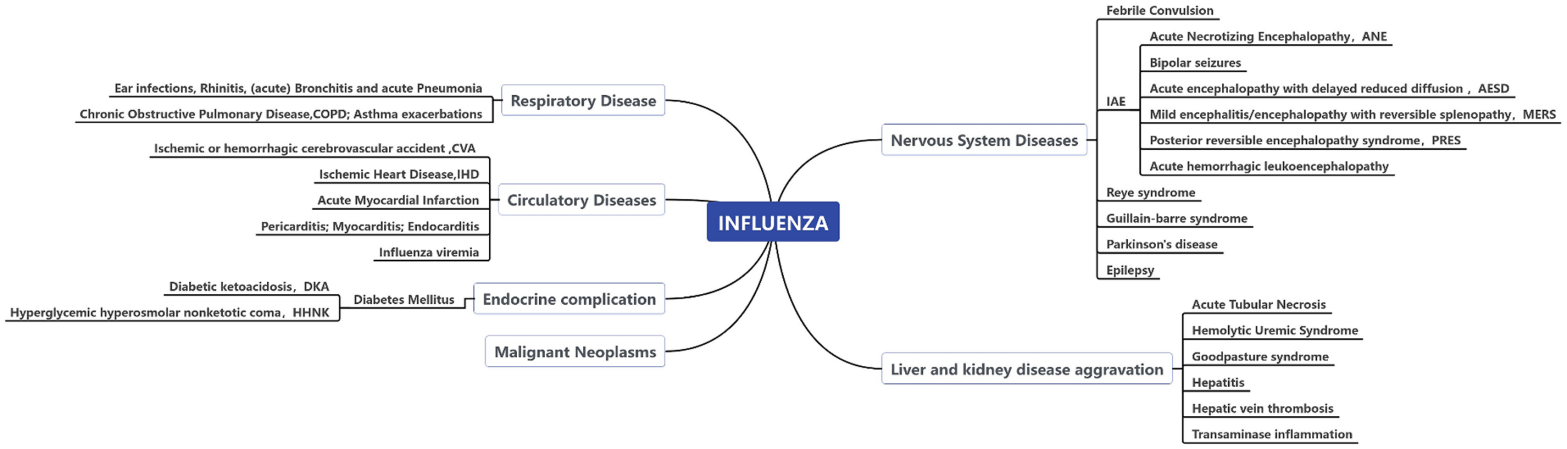
Epidemiological evidence of associated diseases/causes of death selection.

A study estimated the mortality rate of influenza-associated respiratory diseases in China following the 2009 pandemic (5). Several investigations have provided estimates of the burden of mortality in specific cities across China, considering respiratory and circulatory diseases as potential causes of death for estimation purposes (1, 6–16). In recent years, other influenza-associated diseases have been identified as a result of scientific progress. According to literature, influenza has been found to exert a significant impact on malignant tumors (17, 18), diabetes (19–24), and nervous system diseases (25–27). Furthermore, liver cirrhosis and kidney failure are frequent complications of influenza (28–30). Detailed associated diseases are shown in Figure 1. Furthermore, manifestations of influenza may differ as a result of environmental climate changes and social activities. Therefore, it is critical to comprehensively analyze the actual disease burden attributed to influenza in China. Taking into account the diverse climatic zones prevalent across China, it is noteworthy that certain regions exhibit less conspicuous influenza virus activity compared to others. In the selection of model methods, it is pertinent to note the exclusion of approaches such as the rate difference model, the Serfling method, and other techniques reliant on clearly defined seasonal patterns. Therefore, this study employs an alternative method developed by the US Centers for Disease Control, which assumes a proportional relationship between virus activity and deaths caused by influenza (31, 32). Virus surveillance data is utilized to guide the burden model. The objective of this study is to estimate excess mortality caused by influenza within China over the last decade and to verify the disease impact of influenza. The analysis of this indicator is the basis for the country to measure the economic costs of health and thus develop effective policies to avoid future crises.

## 2 Data sources

In 2011, China commenced the enhancement of its nationwide influenza surveillance network in response to the 2009 and 2010 pandemics. In order to ensure stable surveillance conditions, excess influenza mortality data from 2012 to 2021 was selected as the main focus of this study. The annual influenza period was defined as January 1st to December 31st each year. To represent the population accurately, we utilized the average annual population by taking into account the mean population at the end of two consecutive years. Population data were obtained from the statistical yearbook of the National Bureau of Statistics. Weekly data on virus numbers, strain types of influenza, specimen submissions, and proportions of positive subtypes were extracted from etiological surveillance data provided by the Chinese National Influenza Center in order to depict patterns in influenza virus activity. The data spanned from December 2011 to December 2021. We excluded the number of samples co-infected with other viruses.

We extracted surveillance data on mortality and causes from the Disease Surveillance System of China for nine diseases from 2011 to 2021 using ICD-10 codes (Table 1). The diseases included in the data are as follows: all-cause (AC), respiratory system diseases (RD), circulatory system diseases (CD), malignant tumors (MN), diabetes mellitus (DM), nervous system diseases (NSD), chronic liver and kidney diseases (CKLD), ischemic heart disease (IHD), and chronic obstructive pulmonary diseases (COPD). Furthermore, respiratory and circulatory disease indicators were combined as R&C in order to assess their contribution toward excess influenza mortality, in accordance with previous research on influenza disease burden (33). More information on the sources of the data is provided in the Appendix.

**Table 1:** ICD-10 codes for the influenza-associated deaths selected in the Chinese disease surveillance system.

Cause of death	ICD-10 codes
All causes (AC)	
Respiratory diseases (RD)	J00-J99, U07.1
Circulatory diseases (CD)	I00-I99
Malignant neoplasms (MN)	C00-C97
Diabetes mellitus (DM)	E10-E14
Nervous system diseases (NSD)	A39, G00-G99
Chronic liver and kidney disease (CKLD)	K70, K74, N00–N19, Q60
Ischemic heart disease (IHD)	I20-I25
Chronic obstructive pulmonary disease (COPD)	J40-J44
Respiratory and circulatory diseases (R&C)	J00-J99, U07.1, I00-I99

## 3 Methods

Negative binomial regression models were employed to analyze the data on fatalities related to influenza-associated diseases and influenza surveillance utilizing discrete phenomenon tests. The lag effect of influenza virus on outcomes was assessed by incorporating virological data from 0 to 3 weeks prior to mortality into the model, whereas the positive rate of different subtypes served as a proxy for measuring influenza virus activity (27, 34). The fitting equation for the negative binomial regression model, which takes into account different causes of mortality as dependent variables in order to estimate the regression coefficients for various types of influenza viruses, is presented below:

*Y_ti_* = α exp. {β_0_ + β_1_ [t_i_] + β_2_ [t_i_^2^] + β_3_ [t_i_^3^] + β_4_ [sin(2*t_i_π*/12)] + β_5_ [cos(2*t_i_π*/12)] + β_6_ [A (H1N1)_ti_] + β_7_ [A (H3N2)_ti_] + β_8_ [B_ti_]}(1) (31)

Where, *Y*_ti_ represents the number of deaths during *t*_i_ for a specific cause of death, *t*_i_ stands for consecutive months, The term α is the population offset, *β*_0_ is the intercept, *β*_1_ is the linear time trend in months, *β*_2_ and *β*_3_ are non-linear time trends, *β*_4_ and *β*_5_ are seasonal changes in death, and *β*_6_ through *β*_8_ are coefficients associated with the percentages of specimens testing positive for specific influenza viruses in a given month.

The total number of fatalities can be determined by multiplying the regional mortality rate with the corresponding population size. The positive rate of a specific subtype of the influenza virus was determined through the computation of the ratio between the total number of samples examined and the number of samples that tested positive for that particular subtype. Monthly data on positive samples was derived from weekly data (31). The excess mortality rate was determined by calculating the ratio of excess fatalities to the population during the observation period. The total number of excess deaths per month was utilized to calculate the mortality associated with influenza and the overall excess mortality for the entire year (33). The regression coefficient, RR value, ER, and 95% CI of various viruses were calculated to determine the influenza virus with the strongest correlation to death. Furthermore, an analysis of the fluctuating trend was conducted after calculating the percentage of excess mortality and excess fatalities attributable to various causes on an annual average basis. Akaike information criterion (AIC), Bayesian Information Criterion (BIC), and *R*^2^ were used to evaluate the models.

The statistical analysis and data preparation were conducted utilizing SPSS 24.0 and Python 3.11.3 software, respectively.

## 4 Results

We screened out the most relevant lag between different virus subtypes and influenza-related deaths. A (H1N1) virus had a statistically significant effect on 8 causes of death except MN and R&C (*p* < 0.05) (Table 2). With every 1% increase in the positive rate of A (H1N1), the excess risk of influenza-associated deaths in descending order were RD 4.79% (95% CI: 2.04–9.99), COPD 4.04% (95% CI: 1.75–8.23), NSD 2.75% (95% CI, 0.62–7.67), CD 2.08 (0.54–5.16), IHD 1.62 (0.46–3.70), AC 1.03(0.49–1.77), CKLD 0.86 (0.41–1.46), DM 0.10 (−0.10–0.34), and R&C 2.41 (0.95–4.96).

**Table 2:** The influence of virus subtypes on excess deaths from influenza-related diseases.

Type/sub type	Lag (week)	*β*	*S.E.*	RR^a^ (95%CI)	ER^b^ (95%CI)	*P*
AC						
A (H1N1)	0	0.7101	0.1577	2.03 (1.50–2.78)	1.03 (0.49–1.77)	<0.001
A (H3N2)	1	0.0176	0.1383	1.02 (0.78–1.33)	0.02 (−0.22–0.33)	0.898
B	0	0.4799	0.1252	1.62 (1.27–2.07)	0.62 (0.26–1.07)	<0.001
RD						
A (H1N1)	1	1.7554	0.3276	5.79 (2.99–11.38)	4.79 (2.04–9.99)	<0.001
A (H3N2)	1	1.0723	0.2682	2.92 (1.70–5.06)	1.92 (0.73–3.94)	<0.001
B	1	1.0198	0.2559	2.77 (1.70–4.56)	1.77 (0.68–3.58)	<0.001
CD						
A (H1N1)	0	1.1244	0.3543	3.08 (1.54–6.31)	2.08 (0.54–5.16)	0.002
A (H3N2)	2	−0.4347	0.3054	0.65 (0.36–1.17)	−0.35 (−0.64–0.18)	0.155
B	0	0.3344	0.2814	1.40 (0.80–2.47)	0.40 (−0.2–1.43)	0.235
MN						
A (H1N1)	0	0.1247	0.0970	1.13 (0.94–1.37)	0.13 (−0.06–0.37)	0.199
A (H3N2)	1	−0.1271	0.0829	0.88 (0.75–1.04)	−0.12 (−0.25–0.04)	0.125
B	0	0.0017	0.0771	1.00 (0.86–1.17)	0.00 (−0.14–0.17)	0.983
DM						
A (H1N1)	1	0.7881	0.3655	2.20 (1.10–4.48)	0.10 (−0.10–0.34)	0.031
A (H3N2)	2	−0.5366	0.3001	0.58 (0.33–1.04)	−0.42 (−0.68–0.05)	0.074
B	0	0.5863	0.2769	1.80 (1.05–3.13)	0.80 (0.04–2.09)	0.034
NSD						
A (H1N1)	1	1.3226	0.4273	3.75 (1.65–8.79)	2.75 (0.62–7.67)	0.002
A (H3N2)	0	−0.7359	0.3458	0.48 (0.25–0.93)	−0.52 (−0.76–0.06)	0.053
B	0	0.6799	0.3263	1.97 (1.03–3.85)	0.97 (0.04–2.74)	0.037
CKLD						
A (H1N1)	0	0.6218	0.1430	1.86 (1.41–2.47)	0.86 (0.41–1.46)	<0.001
A (H3N2)	1	0.0409	0.1254	1.04 (0.81–1.33)	0.04 (−0.19–0.33)	0.744
B	0	0.5019	0.1136	1.65 (1.32–2.06)	0.65 (0.32–1.06)	<0.001
IHD						
A (H1N1)	0	0.9636	0.2983	2.62 (1.48–4.72)	1.62 (0.46–3.70)	0.001
A (H3N2)	2	−0.2884	0.2619	0.75 (0.46–1.24)	−0.25 (−0.55–0.25)	0.271
B	0	0.7227	0.2369	2.06 (1.30–3.31)	1.06 (0.29–2.28)	0.002
COPD						
A (H1N1)	1	1.6181	0.3086	5.04 (2.72–9.48)	4.04 (1.75–8.23)	<0.001
A (H3N2)	1	1.1901	0.2527	3.29 (1.97–5.52)	2.29 (1.00–4.39)	<0.001
B	1	1.1347	0.2410	3.11 (1.96–4.98)	2.11 (0.94–3.99)	<0.001
R&C						
A (H1N1)	0	1.2256	0.2853	3.41 (1.94–6.08)	2.41 (0.95–4.96)	<0.001
A (H3N2)	2	−0.1616	0.2504	0.85 (0.52–1.39)	−0.15 (−0.48–0.39)	0.519
B	0	0.4910	0.2266	1.63 (1.05–2.57)	0.63 (0.05–1.55)	0.030

^a^Relative risk (RR), RR = exp(β), RR (95% CI) = exp (β ± 1.96 SE). ^b^Excess risk (ER), ER = [exp(β) -1] × 100%, ER (95% CI) = [exp (β ± 1.96 SE) -1] × 100%.

The A (H3N2) virus was found to be exclusively associated with RD and COPD, with a statistically significant correlation (*p* < 0.05). With an increase of 1% in the positive rate of A (H3N2), the excess risk of influenza-associated death was 1.92% (95% CI: 0.73–3.94) and 2.29% (95% CI: 1.00–4.39), respectively, for RD and COPD cases. However, no significant impact on excess mortality due to R&C was observed.

The impact of the B virus on 7 different classes, excluding CD and MN caused by influenza, significantly affects excess deaths (*p* < 0.05). With an increase of 1% in the positive rate of B virus, the excess risk of influenza-associated death in descending order was COPD 2.11% (95% CI: 0.94 ~ 3.99), RD 1.77% (95% CI: 0.68 ~ 3.58), IHD 1.06% (95% CI: 0.29 ~ 2.28), NSD 0.97% (95% CI: 0.04 ~ 2.74), DM 0.80% (95% CI: 0.04 ~ 2.09), CKLD 0.65% (95% CI: 0.32 ~ 1.06), AC 0.62% (95% CI: 0.26 ~ 1.07), and the excess risk for R&C was 0.63% (95% CI: 0.05 ~ 1.55).

A total of 327,520 influenza-positive samples were isolated from 2012 to 2021.

**Figure 2:**
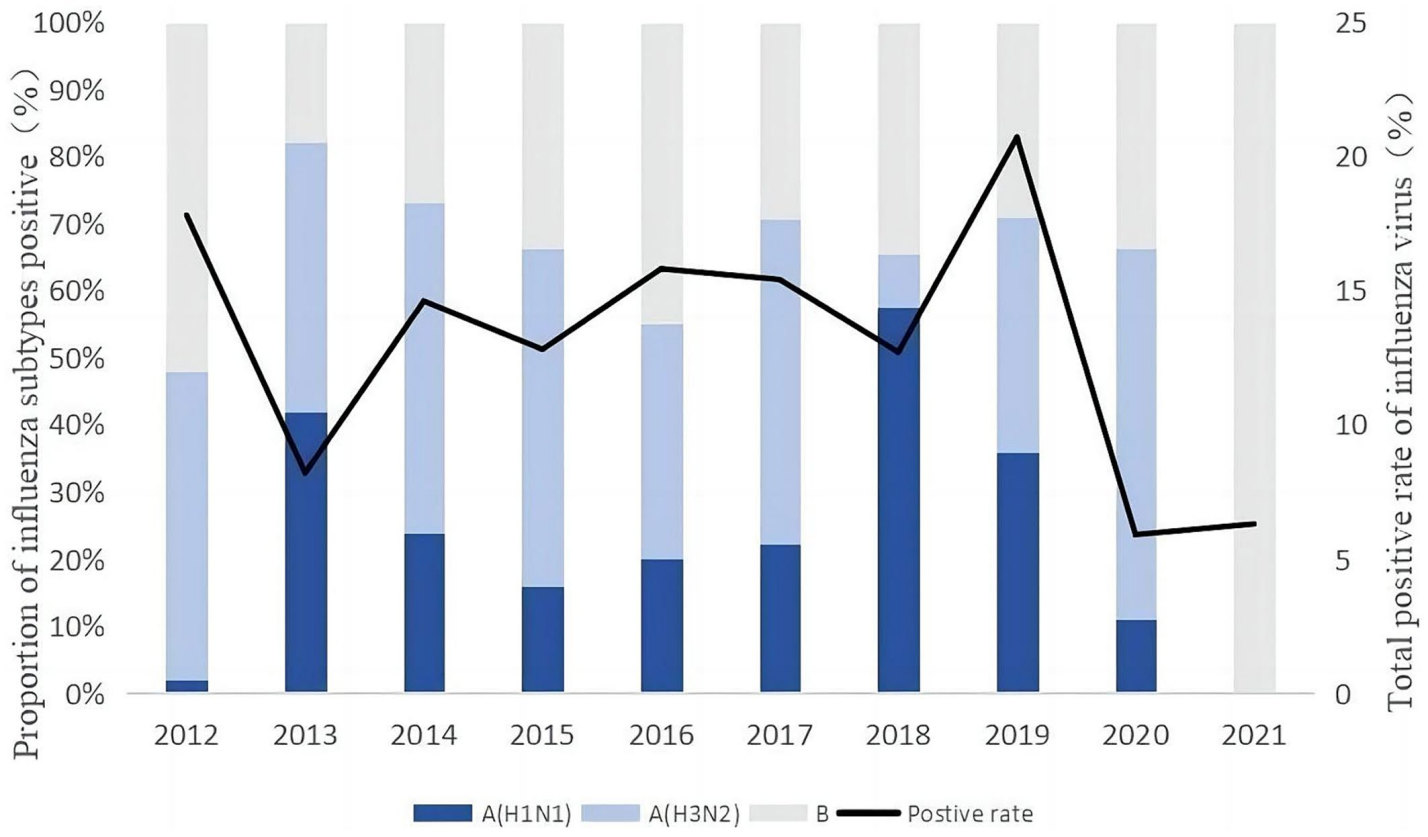
Dominant periods of influenza activity and strain distribution.

Figure 2 illustrates the positive detection rate of influenza virus samples in China among different years. The annual average positive detection rate for influenza virus samples was 13.0%. The year 2019 recorded the highest rate at 20.7%, followed by 2012 at 17.8%, 2016 at 15.8%, 2017 at 15.4%, and 2014 at 14.6%. In contrast, the rates were relatively lower in 2015 (12.8%) and 2018 (12.7%), reaching the lowest point in 2020 at a mere 5.9%. During the research period, a downward trend was observed from 2012 to early 2013. This was followed by an increase until mid-2014, after which a stable fluctuation occurred until late 2018, when an upward trend emerged until late 2019. Subsequently, there was another decline that persisted until late 2020, at which point it reached its lowest level.

Divergences in the prevailing influenza strains were observed among various years in China. Both influenza A (H3N2) and B viruses co-circulated in 2012, 2015, 2016, and 2020. It was influenza A (H1N1) and A (H3N2) in 2013. During 2014 and 2017, Influenza A (H3N2) remained dominant, with a certain proportion (>20%) of A (H1N1) and B virus also present. In 2018, influenza A (H1N1) emerged as the dominant strain, however, Influenza B accounted for a significant positive rate of 35%. In 2019, Influenza A (H1N1), A (H3N2), and B viruses co-circulated, with Influenza B having the lowest positive rate. As for the year 2021, Influenza B virus strains exhibited an absolute advantage.

Data from Table 1 was utilized to fit the negative binomial model. Excess deaths associated with influenza-associated diseases were extrapolated, and the mortality rates were calculated (Table 3). An annual estimate of 201,721.78 cases of influenza-related excess deaths for the entire Chinese population was calculated from 2012 to 2021; these cases accounted for approximately 2.04% of all observed deaths annually. The corresponding excess mortality rate among this population was calculated to be 14.53 per 100,000 individuals.

**Table 3:** Excess influenza-related deaths in China from 2012 to 2021.

Deaths	Average excess deaths^a^	Percentage of excess deaths^b^ (%)	Excess mortality rate of the population^c^ (1/100,000)
AC	201721.78 (126584.26–244324.45)	2.042 (1.281–2.473)	14.526 (9.115–17.594)
RD	51751.82 (32336.24–61525.01)	4.898 (3.060–5.822)	3.727 (2.329–4.430)
CD	63310.96 (39915.88–77294.29)	1.534 (0.967–1.873)	4.559 (2.874–5.566)
DM	4417.27 (2722.13–5295.54)	2.157 (1.329–2.586)	0.318 (0.196–0.381)
NSD	3252.08 (2013.42–3936.86)	2.901 (1.796–3.512)	0.234 (0.145–0.283)
CKLD	3688.68 (2307.71–4458.33)	2.316 (1.449–2.799)	0.266 (0.166–0.321)
IHD	54920.11 (34279.00–66644.27)	3.354 (2.093–4.070)	3.955 (2.468–4.799)
COPD	43311.99 (27019.05–51290.02)	5.454 (3.402–6.458)	3.119 (1.946–3.693)
R&C	117997.43 (74454.51–143431.54)	2.276 (1.436–2.767)	8.497 (5.361–10.328)

^a^The average excess deaths are the difference between the full model prediction and the prediction with the covariate coefficients, which is set to zero for each influenza virus type. The total number of excess deaths per month is used to calculate the excess deaths for the year. ^b^Percentage of excess deaths equal to the average of annual excess deaths divided by the annual number of observed deaths of influenza-associated diseases (%). ^c^The excess mortality rate of the population is equal to the average of annual excess deaths divided by the annual population of China (per 100,000 people per year).

The average annual excess mortality of RD and CD population were, respectively, 3.73 (2.329–4.430) and 4.559 (2.874–5.566). DM, NSD, and CKLD contributed almost equally to influenza-associated excess mortality with rates of 0.318 (0.196–0.381), 0.234 (0.145–0.283), and 0.266 (0.166–0.321). The excess mortality caused by IHD accounted for 86.75% of circulatory diseases, while the excess mortality caused by COPD accounted for 83.69% of respiratory diseases, which were the primary component. In China, the excess mortality rates associated with influenza-induced COPD and IHD were 3.95/100,000 and 3.12/100,000, respectively.

R&C accounted for a significant proportion (58.50%) of these excess deaths over the past decade. Specifically, an average annual occurrence of 117,997.43 influenza-associated excess deaths, accounts for approximately 2.28% of total observed annual deaths attributed to R&C that were observed. Furthermore, the average annual excess mortality rate within this group amounted to approximately 8.50 per 100,000 individuals.

Table 4 shows the models of influenza-related diseases were well fitted. The *R*^2^ value of COPD is the highest, indicating that *X* had the highest explanatory power for *Y* in the model with COPD as the dependent variable. The *R*^2^ value of MN is 0.034. The fitted models of MN deaths were statistically significant, but were not related to the three influenza subtypes, so the fitted deaths may be derived from demographic and seasonal factors. AIC and BIC are mainly applied to model category screening, and are only used as indicators to evaluate the degree of model fitting.

**Table 4:** Model fitting evaluation results.

Dependent variable/evaluation indicator	AIC	BIC	*R* ^2^	*P*
AC	735585.205	735596.355	0.348	<0.001
RD	352561.586	352572.736	0.457	<0.001
CD	1446756.223	1446767.373	0.161	<0.001
MN	63455.412	63466.562	0.034	<0.001
DM	78685.849	78696.999	0.158	<0.001
NMD	58528.775	58539.925	0.171	<0.001
CKLD	12324.269	12335.419	0.370	<0.001
IHD	457279.499	457290.649	0.232	<0.001
COPD	235601.691	235612.841	0.493	<0.001

**Figure 3:**
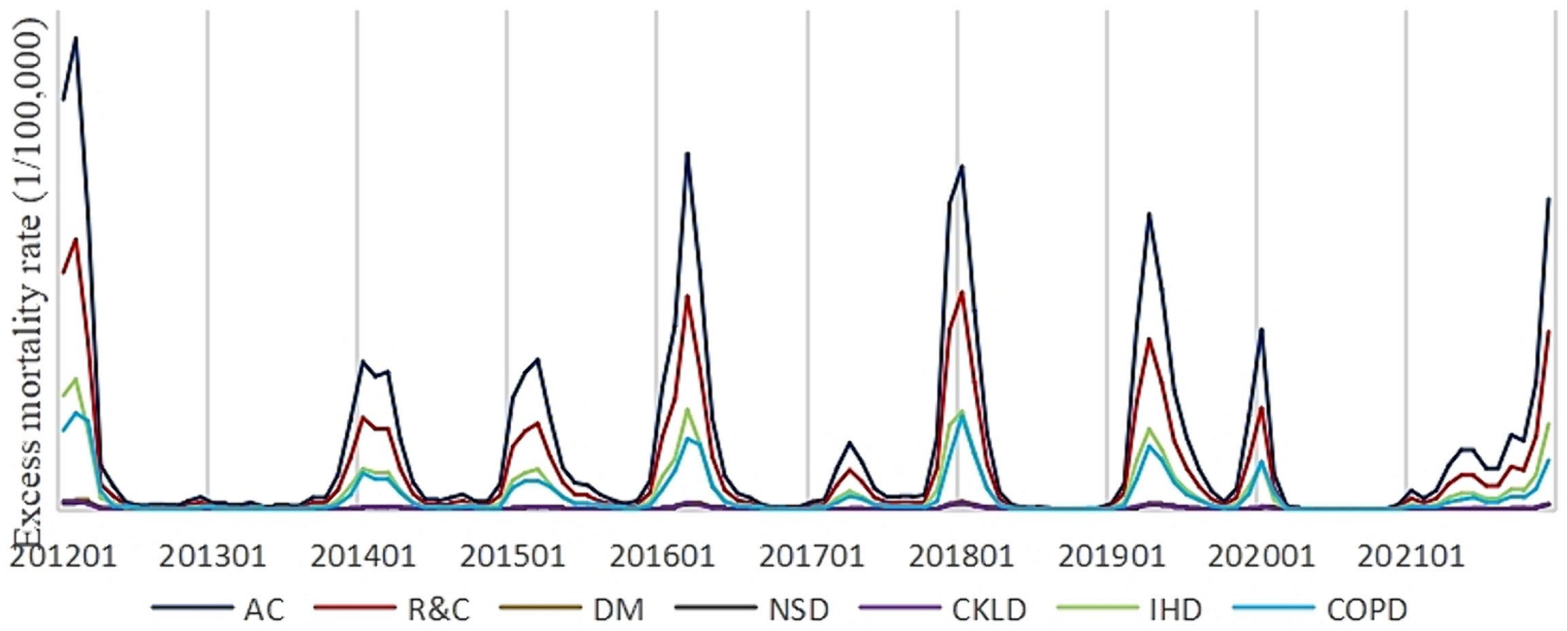
Trends in excess influenza-related deaths by diseases, 2012–2022.

As a result of the correlation between respiratory and circulatory diseases, R&C was utilized as one of the trend analysis indicators. A discernible temporal pattern was observed in excess mortality for respiratory and circulatory diseases, ischemic heart disease, chronic obstructive pulmonary disease, and all causes of death in China between 2012 and 2021. Annual peaks in incidence were consistently observed in January. However, variations were noted among different years regarding the onset month, magnitude, and duration of these mortality peaks (Figure 3).

**Figure 4:**
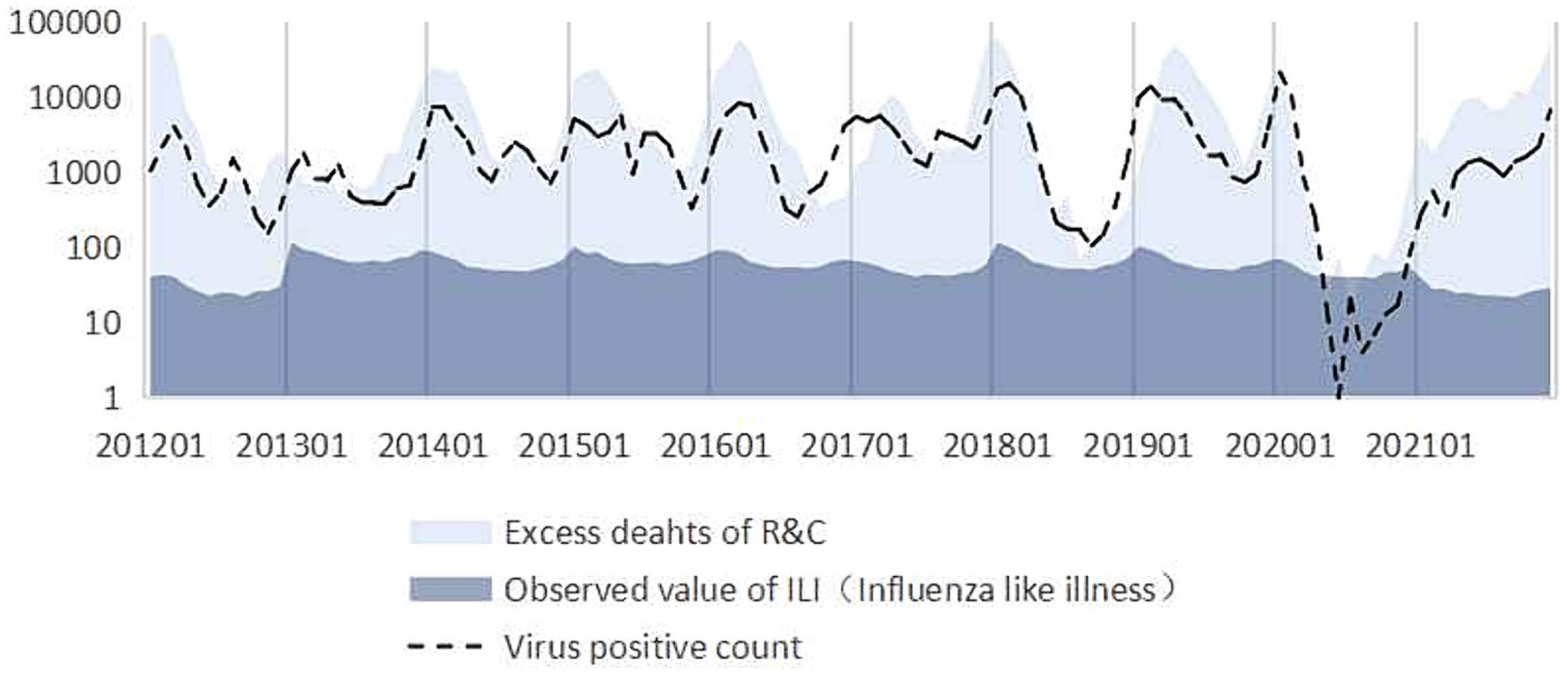
Comparison of virus activity, excess deaths, and surveillance cases of influenza-like illness.

As the observation index, we selected R&C as it exhibited the greatest correlation with excess mortality due to influenza and accounted for the highest proportion. We converted the vertical axis to the logarithmic axis to compare differences. The vertical axis was converted to a logarithmic scale for better comparison of differences (Figure 4). “Observed value of ILI” showed the deaths registered as flu. The “virus positive count” is the number of strains of influenza virus detected by the Influenza Centre. “Excess deaths of R&C” shows the deaths registered as flu and also the deaths which are not registered as flu in respiratory and circulatory diseases.

It can be seen that the trend in excess deaths of R&C estimated by the model is consistent with the trend in virus positives count from 2012 to 2021. However, the excess deaths of R&C are significantly higher than the number of influenza deaths directly detected (Observed value of ILI). And the trend in observed value of ILI does not match the trend of virus positive count.

## 5 Discussion

It is vital to have an accurate assessment of the true impact of influenza epidemics in order to make informed decisions regarding public health. In this study, we examined nine diseases that have been proposed to be associated with influenza in terms of disease burden. Among them, the burden of death from all cause, respiratory diseases, circulatory diseases, diabetes mellitus, nervous system diseases, chronic liver and kidney disease, ischemic heart disease, and chronic obstructive pulmonary disease have been shown to be influenced by the prevalence of influenza viruses. However, based on the statistical data from China spanning the years 2012 to 2021, it was not confirmed that any subtype of influenza had an impact on malignant neoplasms. Influenza A (H3N2) only affected deaths related to respiratory diseases, especially for COPD. Additionally, the influence of the influenza B virus on the burden of circulatory system diseases was also not statistically significant. This may be the result of prior research (5, 33) that linked influenza to disease burdens predominantly using data from pandemic eras, during which economic and social factors significantly influenced causes of death in comparison to stable periods.

Additionally, the annual burden of influenza fluctuates, possibly due to factors such as the prevailing virus in circulation, the efficacy of vaccines, and the duration of the annual influenza epidemic. A significant decrease in influenza virus activity was observed during the post-pandemic period following the A (H1N1) and COVID-19 pandemics. This decline was accompanied by changes in dominant viral strains, indicating the potential efficacy of interventions such as vaccination and reduced population susceptibility.

The average annual excess mortality rate for the entire population in China due to all causes (AC) was estimated at 14.53 per 100,000 individuals from 2012 to 2021. R&C remained the leading cause of death in influenza virus circulation, accounting for 58.50% of all deaths, with an excess mortality rate of 8.497 per 100,000 individuals. Previous research has primarily focused on the burden of respiratory and circulatory diseases; however, more influenza-associated diseases have been identified over the past decade. The mortality figures presented in this paper are consistent with those reported in a global study that utilized data from prior years to estimate the aggregate mortality (1). Over the past decade, the average annual excess deaths and population mortality of AC and R&C have decreased in comparison to the influenza-associated excess deaths in China from 2004 to 2009 (33). This decline is likely due to enhanced infectious disease prevention measures such as vaccines.

However, the influenza percentage of excess deaths of respiratory and circulatory diseases has increased. This is because influenza usually causes acute episodes of respiratory and circulatory diseases. In recent years, medical advances have reduced the overall number of deaths from respiratory and circulatory diseases, and increased the cure rate for chronic respiratory and circulatory diseases in particular. The proportion of deaths caused by influenza has increased. COPD and IHD significantly contribute to the excess mortality burden associated with influenza in the Respiratory and circulatory systems. Furthermore, the results of this study revealed that seasonal influenza during stable periods also contributed to a certain proportion of the excess mortality burden for patients with chronic liver and kidney disease, diabetes mellitus, and diseases of the nervous system. Therefore, it is imperative to implement effective preventive measures that target these vulnerable populations. The model evaluation results showed that the models of influenza-related diseases were well fitted.

The distribution of influenza virus in China exhibited a consistent pattern throughout the year, from 2012 to 2021. In contrast to diseases that were closely associated with AC and R&C, which exhibited negligible seasonal variations, the excess fatalities caused by these conditions in China peaked in the winter and spring. It was previously believed that the influenza virus in China was most prevalent during the winter and disappeared during the summer (27). This is most likely the result of monitoring only the most immediate causes of flu deaths. In addition, the results of this study indicate that the manifestations of influenza were evenly distributed and more manifested as non-R&C causes of death during the summer and autumn. Therefore, influenza surveillance during the summer is more complicated, and the scope of surveillance should be larger. The distribution of excess deaths attributed to influenza-related diseases under different conditions will be analyzed in subsequent research.

The trend of influenza-associated excess deaths related to R&C was consistent with the pattern of virus activity, thereby validating the findings of this research. However, it is critical to acknowledge that the actual surveillance value was significantly lower than the estimated value. Consequently, accurately capturing the mortality burden caused by seasonal influenza through epidemiological surveillance based solely on case notification proves challenging. These deficiencies result in a significant underestimate of the actual impact of the disease. Therefore, it becomes imperative to employ appropriate methodologies for predicting epidemic indicators of infectious diseases.

## 6 Limitations

This study only included cause of death and death data within the scope of China’s disease surveillance system. Diseases related to influenza, such as hematopoietic immune diseases, were not included in this study because the mortality rate collected was too low and statistical data were not available every year. Relevant epidemiological data will be collected separately for analysis in the next phase of the study. Although the data is nationally representative and the current disease surveillance system in China adopts the sentinel surveillance mode, it does not encompass the entire population and cannot be correspond to virus strain surveillance sentinels. Therefore, its sole purpose is to estimate the overall disease burden in China. Differences in demographic characteristics were analyzed based on hospital case samples in another study.

## 7 Conclusion

We conducted an analysis of the excess mortality resulting from the influenza virus in China over the past decade. Our investigation identified and categorized the different types of excess mortality caused by influenza, with a particular focus on estimating the proportion of excess mortality attributable to causes other than respiratory and circulatory death. These indicators served as a scientific foundation for the current optimization of influenza surveillance and early warning systems in China.

## Data availability statement

The raw data supporting the conclusions of this article will be made available by the authors, without undue reservation.

## Author contributions

T-LY: Conceptualization, Data curation, Writing – original draft. NC: Data curation, Formal analysis, Writing – review & editing. J-YZ: Data curation, Formal analysis, Writing – review & editing. SY: Data curation, Formal analysis, Writing – review & editing. W-ML: Data curation, Formal analysis, Writing – review & editing. X-HG: Data curation, Formal analysis, Writing – original draft. H-LS: Data curation, Formal analysis, Writing – review & editing. H-PH: Data curation, Formal analysis, Writing – review & editing.

## References

[1] Iuliano AD Roguski KM Chang HH Muscatello DJ Palekar R Tempia S. Global seasonal influenza-associated mortality collaborator network. Estimates of global seasonal influenza-associated respiratory mortality: a modelling study. Lancet. (2018) 391:1285–300. doi: 10.1016/S0140-6736(17)33293-2, PMID: 29248255 PMC5935243

[2] World Health Organization. (2019). Fact sheet on influenza (seasonal) [EB/OL]. [2019-03-20].

[3] Woyessa AB Mengesha M Belay D Tayachew A Ayele W Beyene B. Epidemiology of influenza in Ethiopia: findings from influenza sentinel surveillance and respiratory infection outbreak investigations, 2009-2015. BMC Infect Dis. (2018) 18:449. doi: 10.1186/s12879-018-3365-5, PMID: 30176806 PMC6122732

[4] Thompson WW Comanor L Shay DK. Epidemiology of seasonal influenza: use of surveillance data and statistical models to estimate the burden of disease. J Infect Dis. (2006) 194:S82–91. doi: 10.1086/507558 17163394

[5] Li L Liu Y Wu P Peng Z Wang X Chen T. Influenza-associated excess respiratory mortality in China, 2010-15: a population-based study. Lancet Public Health. (2019) 4:e473–81. doi: 10.1016/S2468-2667(19)30163-X, PMID: 31493844 PMC8736690

[6] Wu S Wei Z Greene CM Yang P Su J Song Y. Mortality burden from seasonal influenza and 2009 H1N1 pandemic influenza in Beijing, China, 2007-2013. Influenza Other Respir Viruses. (2018) 12:88–97. doi: 10.1111/irv.12515, PMID: 29054110 PMC5818349

[7] Zhang H Xiong Q Wu P Chen Y Leung NHL Cowling BJ. Influenza-associated mortality in Yancheng, China, 2011-15. Influenza Other Respir Viruses. (2018) 12:98–103. doi: 10.1111/irv.12487, PMID: 29193690 PMC5818359

[8] Liu XX Qin G Li X Zhang J Zhao K Hu M. Excess mortality associated with influenza after the 2009 H1N1 pandemic in a subtropical city in China, 2010-2015. Int J Infect Dis. (2017) 57:54–60. doi: 10.1016/j.ijid.2017.01.039, PMID: 28167255

[9] Yu X Wang C Chen T Zhang W Yu H Shu Y. Excess pneumonia and influenza mortality attributable to seasonal influenza in subtropical Shanghai, China. BMC Infect Dis. (2017) 17:756. doi: 10.1186/s12879-017-2863-1, PMID: 29212467 PMC5719671

[10] Huang ZF Liu XJ Wu YS Yang LP Zou YH Li Y. Application of Serfling regression model in estimating excess mortality from influenza in Shenzhen. Chin J Dis Cont. (2017) 21:1170–4. doi: 10.16462/j.cnki.zhjbkz.2017.11.022

[11] Guo RN Zheng HZ Ou CQ Huang LQ Zhou Y Zhang X. Impact of influenza on outpatient visits, hospitalizations, and deaths by using a time series Poisson generalized additive model. PLoS One. (2016) 11:e0149468. doi: 10.1371/journal.pone.0149468 26894876 PMC4760679

[12] Lao XY Jiao SL Ji W Yi B. Analysis of excess death related to influenza in Ningbo city. Prev Med. (2016) 28:1010–3. doi: 10.19485/j.cnki.issn1007-0931.2016.10.010

[13] Wang H Fu C Li K Lu J Chen Y Lu E. Influenza associated mortality in southern China, 2010-2012. Vaccine. (2014) 32:973–8. doi: 10.1016/j.vaccine.2013.12.013, PMID: 24370709

[14] Li WJ Wang DY. Research progress of influenza burden in China. Chin J Zoonoses. (2019) 35:928–33.

[15] Roth GA Johnson C Abajobir A Abd-Allah F Abera SF Abyu G. Global, regional, and National Burden of cardiovascular diseases for 10 causes, 1990 to 2015. J Am Coll Cardiol. (2017) 70:1–25. doi: 10.1016/j.jacc.2017.04.052, PMID: 28527533 PMC5491406

[16] Blackburn R Zhao H Pebody R Hayward A Warren-Gash C. Laboratory-confirmed respiratory infections as predictors of hospital admission for myocardial infarction and stroke: time-series analysis of English data for 2004-2015. Clin Infect Dis. (2018) 67:8–17. doi: 10.1093/cid/cix1144, PMID: 29324996 PMC6005111

[17] Bosaeed M Kumar D. Seasonal influenza vaccine in immunocompromised persons. Hum Vaccin Immunother. (2018) 14:1311–22. doi: 10.1080/21645515.2018.1445446, PMID: 29485353 PMC6037456

[18] Bitterman R Eliakim-Raz N Vinograd I Zalmanovici Trestioreanu A Leibovici L Paul M. Influenza vaccines in immunosuppressed adults with cancer. Cochrane Database Syst Rev. (2018) 2:CD008983. doi: 10.1002/14651858.CD008983.pub3, PMID: 29388675 PMC6491273

[19] Hulme KD Gallo LA Short KR. Influenza virus and glycemic variability in diabetes: a killer combination? Front Microbiol. (2017) 8:861. doi: 10.3389/fmicb.2017.00861, PMID: 28588558 PMC5438975

[20] Moghadami M Honarvar B Sabaeian B Zamiri N Pourshahid O Rismanchi M. H1N1 influenza infection complicated with diabetic ketoacidosis. Arch Iran Med. (2012) 15:55–8. PMID: 22208446

[21] Cano M Iglesias P Pérez G Díez JJ. Infección por virus influenza A (H1N1) como causa de cetoacidosis diabética grave en la diabetes tipo 1 [Influenza A virus (H1N1) infection as a cause of severe diabetic ketoacidosis in type 1 diabetes]. Endocrinol Nutr. (2010) 57:37–8. doi: 10.1016/S1575-0922(10)70008-5 20172486

[22] Samson SI Konty K Lee WN Quisel T Foschini L Kerr D. Quantifying the impact of influenza among persons with type 2 diabetes mellitus: a new approach to determine medical and physical activity impact. J Diabetes Sci Technol. (2021) 15:44–52. doi: 10.1177/1932296819883340, PMID: 31747789 PMC7780362

[23] Klekotka RB Mizgała E Król W. The etiology of lower respiratory tract infections in people with diabetes. Pneumonol Alergol Pol. (2015) 83:401–8. doi: 10.5603/PiAP.2015.0065 26379004

[24] Pearson-Stuttard J Blundell S Harris T Cook DG Critchley J. Diabetes and infection: assessing the association with glycaemic control in population-based studies. Lancet Diabetes Endocrinol. (2016) 4:148–58. doi: 10.1016/S2213-8587(15)00379-4, PMID: 26656292

[25] Ekstrand JJ. Neurologic complications of influenza. Semin Pediatr Neurol. (2012) 19:96–100. doi: 10.1016/j.spen.2012.02.004 22889537

[26] Sanz Fadrique R Martín Arias L Molina-Guarneros JA Jimeno Bulnes N García OP. Guillain-Barré syndrome and influenza vaccines: current evidence. Rev Esp Quimioter. (2019) 32:288–95. PMID: 31232571 PMC6719653

[27] Wong CM Chan KP Hedley AJ Peiris JS. Influenza-associated mortality in Hong Kong. Clin Infect Dis. (2004) 39:1611–7. doi: 10.1086/425315, PMID: 15578360

[28] Leung VKY Wong JY Barnes R Kelso J Milne GJ Blyth CC. Excess respiratory mortality and hospitalizations associated with influenza in Australia, 2007-2015. Int J Epidemiol. (2022) 51:458–67. doi: 10.1093/ije/dyab138, PMID: 34333637

[29] Eder M Omic H Gorges J Badt F Kikic Z Saemann MD. Influenza vaccination uptake and factors influencing vaccination decision among patients with chronic kidney or liver disease. PLoS One. (2021) 16:e0249785. doi: 10.1371/journal.pone.0249785, PMID: 33848305 PMC8043408

[30] Watanabe T. Renal complications of seasonal and pandemic influenza a virus infections. Eur J Pediatr. (2013) 172:15–22. doi: 10.1007/s00431-012-1854-x, PMID: 23064728

[31] Thompson WW Shay DK Weintraub E Brammer L Bridges CB Cox NJ. Influenza-associated hospitalizations in the United States. JAMA. (2004) 292:1333–40. doi: 10.1001/jama.292.11.1333 15367555

[32] Thompson WW Shay DK Weintraub E Brammer L Cox N Anderson LJ. Mortality associated with influenza and respiratory syncytial virus in the United States. JAMA. (2003) 289:179–86. doi: 10.1001/jama.289.2.179 12517228

[33] Sa L. Study on excess influenza-related death burden at provincial level in China from 2004 to 2009[D]. Chinese Center for Disease Control and Prevention (2016).

[34] Zhang WZ Chen DN Shi JX Wu D Zhang JS. Estimation of excess deaths related to influenza in Shunyi District, Beijing, 2010–2015. Public Health Prevent Med. (2016) 27:26–30.

