# Supplementary material for: Excess multi-cause mortality linked to influenza virus infection in China, 2012–2021: a population-based study
Source: Front Public Health. 2024 May 30;12:1399672. doi: 10.3389/fpubh.2024.1399672 (PMC11182332; doi:10.3389/fpubh.2024.1399672)
Supplement: Supplementary file 1 [file Data_Sheet_1.docx]

**[Excess Multi-Cause Mortality Linked to Influenza Virus Infection in China, 2012–2021: A Population-based Study](https://pubmed.ncbi.nlm.nih.gov/31493844/)**

Appendix

1 Supplementary information of Chinese National Influenza Center

Influenza etiology data for this study were collected from the influenza surveillance system of the Chinese National Influenza Center.After the Influenza A (H1N1) pandemic in 2009-2010, China further expanded the surveillance network covered by the Chinese National Influenza Center.All sentinel hospitals and laboratories of the influenza surveillance Network carry out influenza-like case surveillance throughout the year.The etiological monitoring method has also been optimized for the more sensitive method of Reverse Transcription- Polymerase Chain Reaction (RT-PCR)[1].Up to now, the data of influenza laboratories came from 556 influenza surveillance sentinel hospitals and 411 network laboratories, covering all cities and key counties in China.Sentinel hospitals are located in 15 provinces in southern China:Shanghai,Jiangsu, Zhejiang, Anhui, Fujian, Jiangxi, Hubei, Hunan, Guangdong, Guangxi, Hainan, Chongqing, Sichuan, Guizhou, Yunnan.And 16 provinces in northern China: Beijing, Tianjin, Hebei, Shanxi, Inner Mongolia, Liaoning, Jilin, Heilongjiang, Shandong, Henan, Tibet, Shaanxi, Gansu, Qinghai, Ningxia, Xinjiang.Influenza surveillance cases are defined as those with fever (body temperature ≥38°C), accompanied by cough or sore throat.The time of fever should be within the course of this acute fever, and the identification of body temperature includes the self-measurement of body temperature by the patient and the detection of body temperature by the medical institution.The influenza monitoring calendar is arranged according to ISO8601.Influenza surveillance Network laboratories shall identify influenza virus subtypes or strains within 3 working days after receiving specimens collected by sentinel hospitals using nucleic acid testing methods.For samples that test positive, virus isolation using MDCK cells in good condition and/or SPF chicken embryos is required within 1 week[2-3].

2 Supplementary information of Disease Surveillance Points System[4]

The current surveillance dataset of the National Disease Surveillance Point System has been updated to the 2021 version.The number of monitoring sites reached 605, with a total monitoring population of more than 300 million, accounting for about 24% of the country's population.The system is based on the principle of stratified cluster random sampling, in order to ensure the high quality and high reliability of the monitoring system data.Before the data analysis results were released, the data quality was evaluated.Data from some monitoring sites considered to be seriously under-reported and likely to affect the overall results were excluded. The mortality rate of monitoring sites was less than 4.5‰ as the exclusion criterion, and the lowest mortality rate of the monitoring sites included in the analysis was 4.51‰.

Reference

[1] Li Sa. Study on excess influenza-related death burden at provincial level in China from 2004 to 2009 [D]. Chinese Center for Disease Control and Prevention,2015.

[2] Notice of the Chinese Center for Disease Control and Prevention on Issuing National Technical Guidelines for Influenza Surveillance (2017 edition) https://ivdc.chinacdc.cn/cnic/zyzx/jcfa/201709/P020170930331067634607.pdf

[3] Li L, Liu Y, Wu P, Peng Z, Wang X, Chen T, Wong JYT, Yang J, Bond HS, Wang L, Lau YC, Zheng J, Feng S, Qin Y, Fang VJ, Jiang H, Lau EHY, Liu S, Qi J, Zhang J, Yang J, He Y, Zhou M, Cowling BJ, Feng L, Yu H. Influenza-associated excess respiratory mortality in China, 2010-15: a population-based study. Lancet Public Health. 2019 Sep;4(9):e473-e481.

[4] Chinese Center for Disease Control and Prevention-Monitoring and data.Information issue.https://ncncd.chinacdc.cn/jcysj/siyinjcx/
